# Retraction: Long noncoding RNA DLEU1 promotes cell proliferation and migration of Wilms tumor through the miR-300/HOXC8 axis

**DOI:** 10.1039/d1ra90051j

**Published:** 2021-01-27

**Authors:** Laura Fisher

**Affiliations:** Royal Society of Chemistry Thomas Graham House, Science Park, Milton Road Cambridge CB4 0WF UK advances-rsc@rsc.org

## Abstract

Retraction of ‘Long noncoding RNA DLEU1 promotes cell proliferation and migration of Wilms tumor through the miR-300/HOXC8 axis’ by Wen’an Ge *et al.*, *RSC Adv.*, 2019, **9**, 40240–40247, DOI: 10.1039/C9RA07215B.

The Royal Society of Chemistry hereby wholly retracts this *RSC Advances* article due to concerns with the reliability of the data. The paper was analysed by experts who fact-checked the identities of the described nucleotide sequence reagents,^1^ and found errors with the following nucleotide sequence reagents reported in the article: HOXC8 forward and reverse primers, and miR-300 reverse primer. The HOXC8 primers would not be expected to bind to nor amplify the target sequence, and therefore the results in Fig. 6 are unreliable. The reported miR-300 reverse primer is non-targeting and does not bind to miR-300, therefore the results shown in Fig. 4 and 5 are not reliable.

Given the significance of the concerns about the validity of the data, the findings presented in this paper are not reliable.

The authors have been informed but have not responded to any correspondence regarding the retraction.

Signed: Laura Fisher, Executive Editor, *RSC Advances*.

Date: 15^th^ January 2021.

## Supplementary Material
